# Supplementary figures and images for: Multiscale representations of community structures in attractor neural networks
Source: PLoS Comput Biol. 2021 Aug 23;17(8):e1009296. doi: 10.1371/journal.pcbi.1009296 (PMC8412329; doi:10.1371/journal.pcbi.1009296)

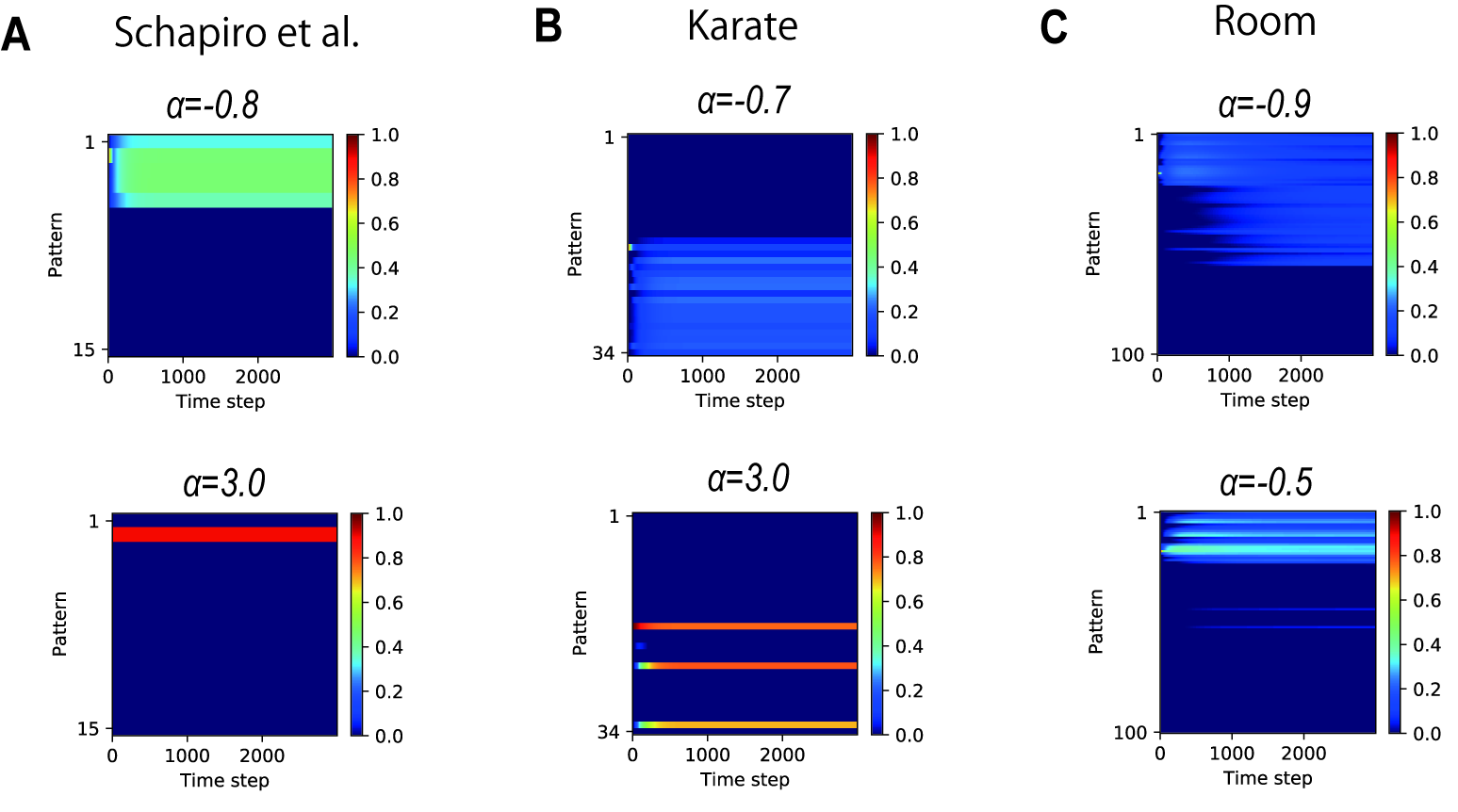

Supplement: S1 Fig — (TIF) [file pcbi.1009296.s001.tif]

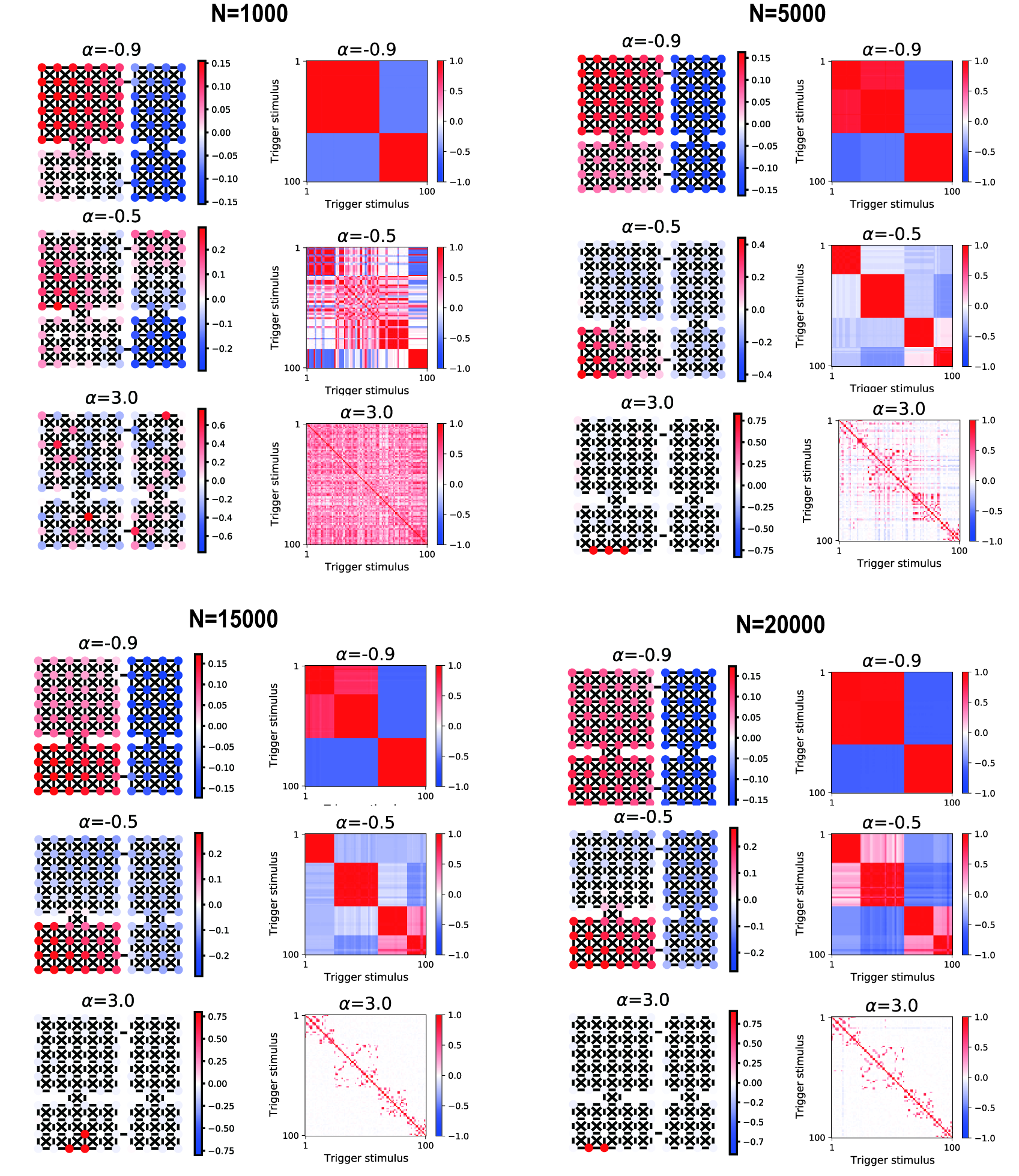

Supplement: S2 Fig — Pattern overlaps of example attractors (left) and pattern correlation matrices (right). (TIF) [file pcbi.1009296.s002.tif]

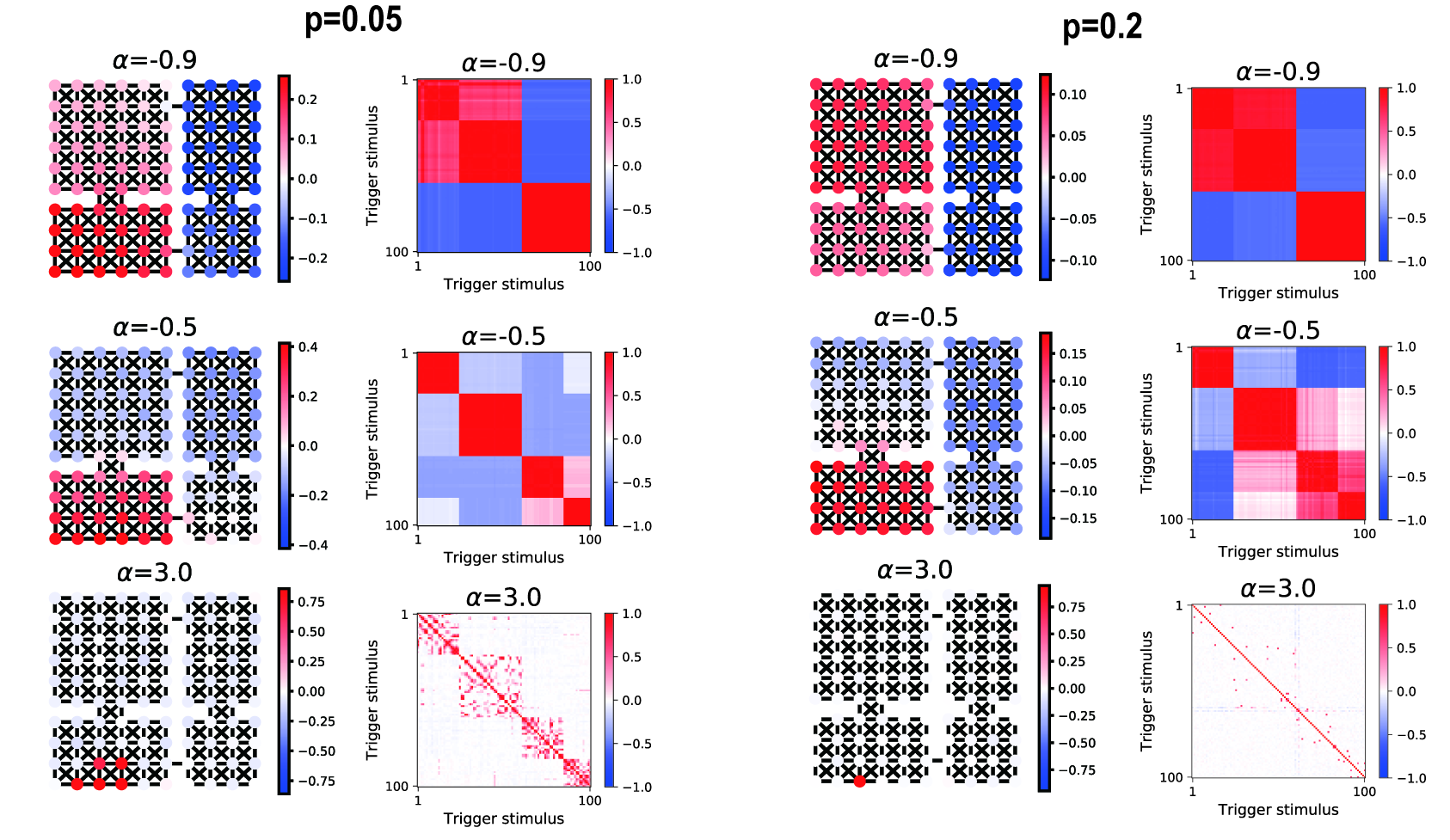

Supplement: S3 Fig — Pattern overlaps of example attractors (left) and pattern correlation matrices (right). (TIF) [file pcbi.1009296.s003.tif]

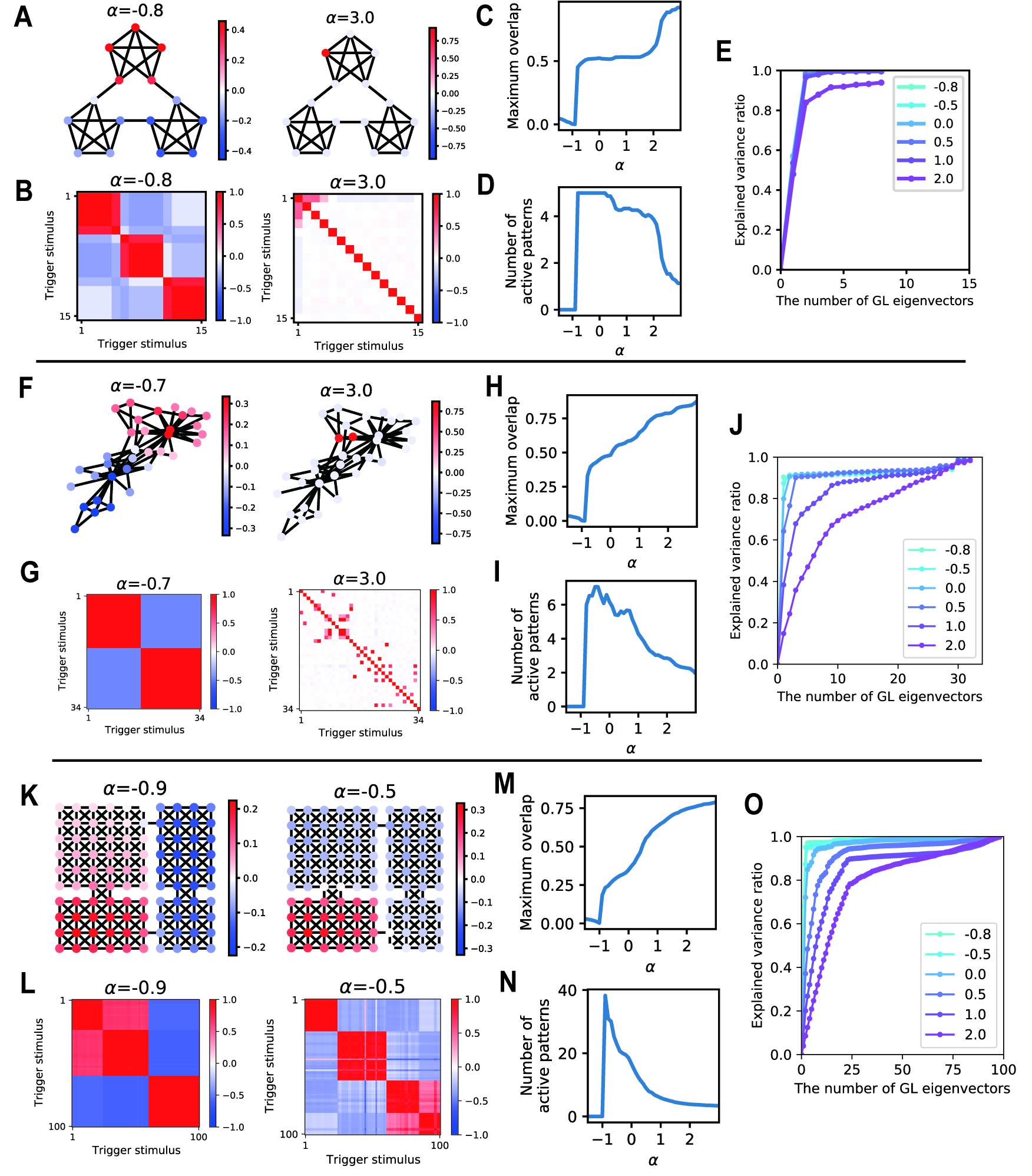

Supplement: S4 Fig — (A) Pattern overlaps of example attractor patterns. (B) Correlation matrices between activity patterns in the attractor states reached from different trigger stimuli (nodes). (C) Maximum pattern overlaps obtained by various values of α. (D) Numbers of active patterns obtained by various values of α. (E) The ratio of variance of overlap distributions explained by various number of graph Laplacian eigenvectors. The color indicates the value of α. We note that, in C-E, we averaged values from all attractors reached from different trigger stimuli. (F-J) Results for Karate-club network. (K-O) Results for compartmentalized rooms. (TIF) [file pcbi.1009296.s004.tif]

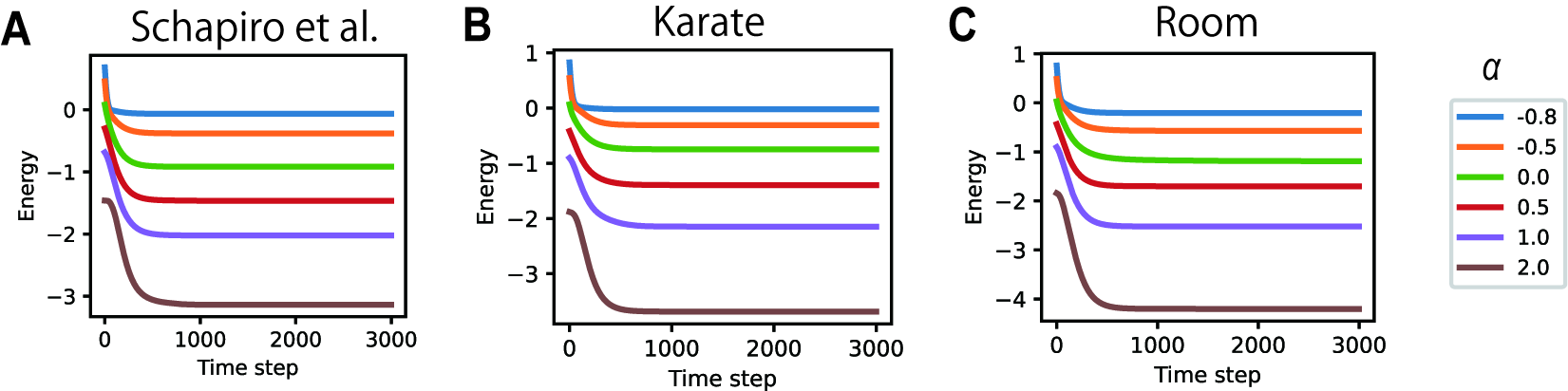

Supplement: S5 Fig — (A) The graph used in Schapiro et al. (2013). (B)Karate club network. (C) The four-room graph. (TIF) [file pcbi.1009296.s005.tif]

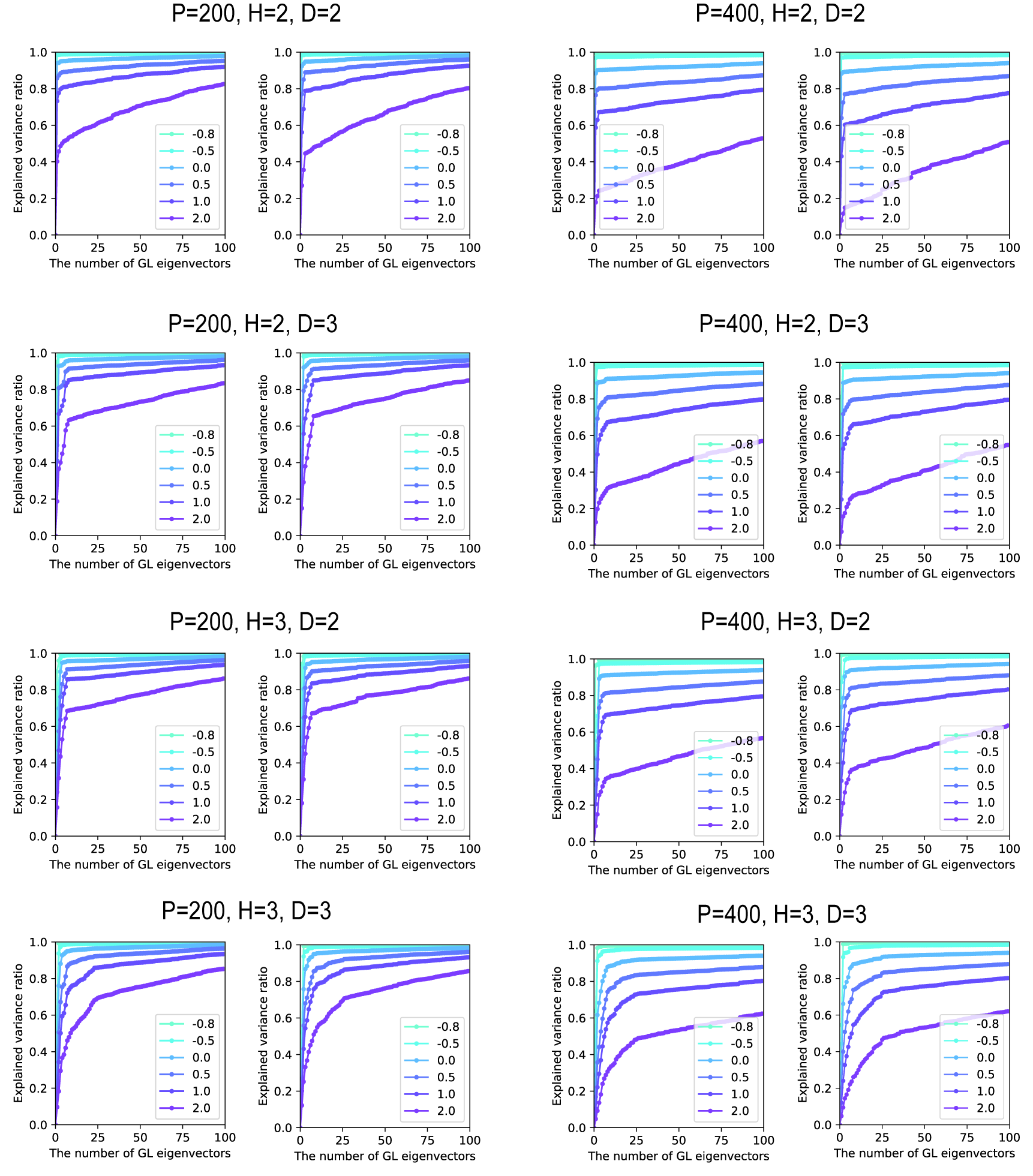

Supplement: S6 Fig — P, H, D are the number of nodes, the number of hierarchy, the number of division in each hierarchy, respectively, Two plots in each setting show results from two different random seeds (different link structures and different memory patterns). (TIF) [file pcbi.1009296.s006.tif]

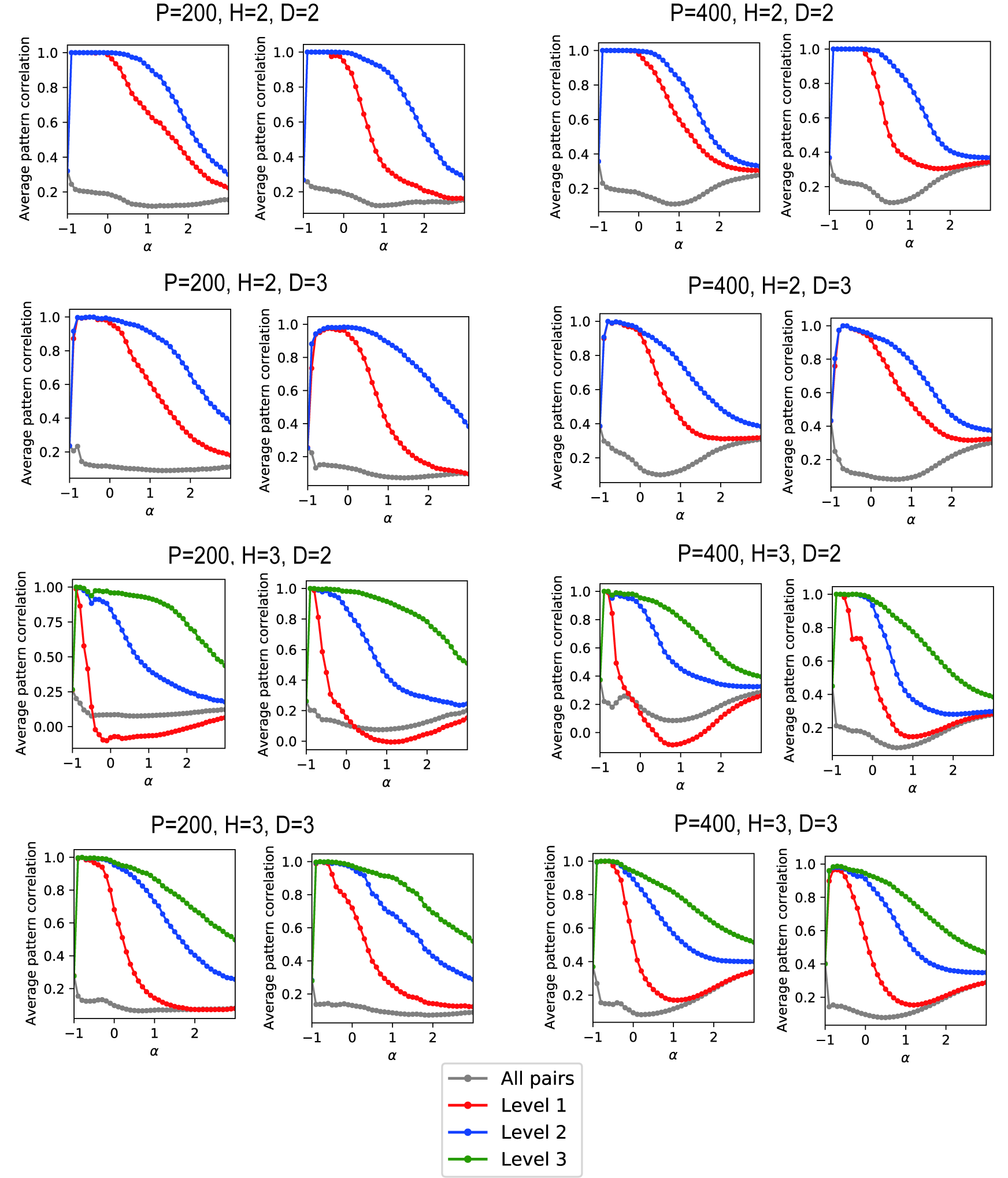

Supplement: S7 Fig — P, H, D are the number of nodes, the number of hierarchy, the number of division in each hierarchy, respectively, Two plots in each setting show results from two different random seeds (different link structures and memory patterns). A level-h pair is in a same community in level h and not in a same community in level (h+1) (the latter condition was not applied if h = H). (TIF) [file pcbi.1009296.s007.tif]

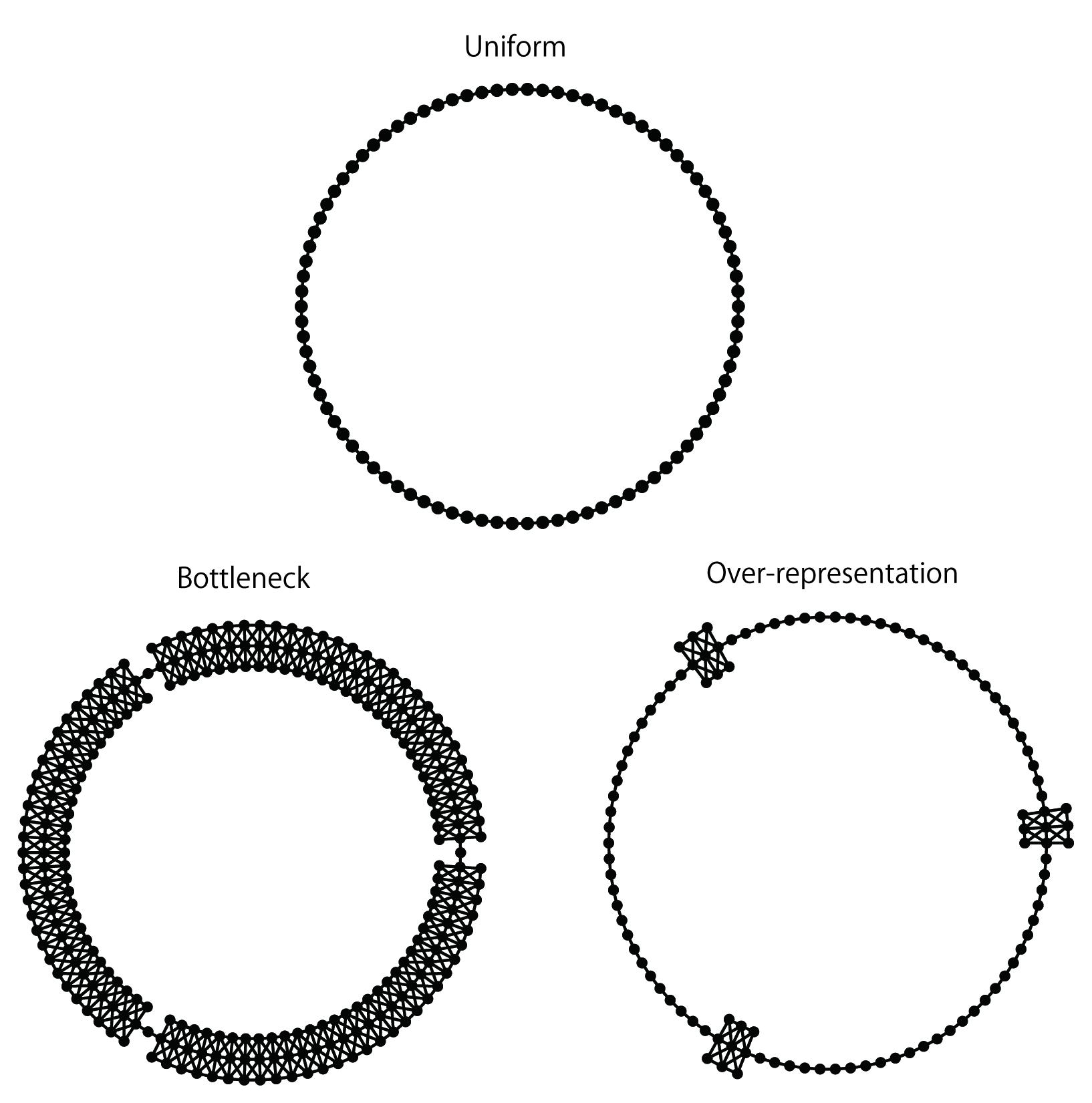

Supplement: S8 Fig — (TIF) [file pcbi.1009296.s008.tif]
