# Supplementary material for: Multiscale representations of community structures in attractor neural networks
Source: PLoS Comput Biol. 2021 Aug 23;17(8):e1009296. doi: 10.1371/journal.pcbi.1009296 (PMC8412329; doi:10.1371/journal.pcbi.1009296)
Supplement: Attachment — Submitted filename: response_to_reviewers.pdf [file pcbi.1009296.s009.pdf]

Response to reviewers

We would like to thank all reviewers for careful reading of our manuscript and valuable comments on it. Following the review comments, we have added novel simulations results and improved descriptions in our manuscript. Following the recommended format of PLOS Comp Biol, we have included the figure legends in the main text of the revised manuscript. Furthermore, we asked a professional proofreading service to check our manuscript before the resubmission.

Below is our point-to-point reply. In the response, we specified revised parts by line numbers in the marked-up copy (not the clean version of the revised manuscript).

Reviewer #1: This work by authors Tatsuya Haga and Tomoki Fukai is both interesting and elegant. The work studies a generalized Hopfield network (called LAM), capable of generating segmented representations of the stored memory patterns. The authors show the link between LAM and graph-theoretic methods. I would recommend the publication of this work, when some minor points are clarified (listed below).

Reply - We thank the reviewer for the positive comments on our manuscript.

1. Hebbian learning is mentioned, and some derivations are made in the methods, however is it actually used in the simulations? If it is not used, am I correct to say then that H is hardcoded? Did you verify using simulations whether Hebbian learning as written in equation 8 would lead to the correct weight structure when doing a random walk on the graph?

Reply – Our simulations were based solely on the hard-coded synaptic weights shown in Eq. (2) (which is an extension of the Hebbian learning paradigm of conventional Hopfield model), and we did not perform on-line learning of weights from random walk on the graph. To avoid confusion, we have deleted the specific learning rule pointed out by the reviewer. We are currently working on a new project in which we use our model in reinforcement learning problems and have partially succeeded on-line learning of synaptic weights. We wish to report these results elsewhere.

2. I have some trouble understanding going from equation 10 on line 457 to the

equation on line 460. Could you provide some more explanation?

Reply – As mentioned above, we have deleted the corresponding part including Eq. (10).

3. How do you choose alpha_max? Is this a constant you set for each type of graph or the result of some derivation?

Reply – Although alpha_max is necessary for biological interpretations of synaptic weights, the parameter is mathematically redundant and its value does not affect the model's behaviors. Therefore, we did not use its specific value anywhere in the present simulations. We have clarified these points in the Methods section of the revised manuscript (L736 – L738).

4. It might be helpful for the reader to show pattern overlaps as a function of simulated time steps for the graphs in figure 2 (for example in a suppl figure?). Essentially like you also did figure 5B. This is a suggestion only of course.

Reply – We have added such plots in S1 Fig. of the revised manuscript.

5. Line 550-551: only one of those conditions should be valid? What does it mean when the overlap is 0.05.

Reply – We counted the number of patterns that satisfied both two conditions but not only one of them. The detection threshold for the pattern overlap can be arbitrary. However, we chose the threshold value of 0.05 as this value enables us to detect pattern activation at $\alpha \approx$-0.9 but avoid detecting noisy patterns when $\alpha \leq -1$. Removal of this condition does not significantly change the results in other parameter regions.

6. Line 126-127 is confusing to me. In the first half of the sentence you write that the weights are non-negative but in the second part of the sentence the range is from -1 (negative) to alpha_max. Also, what is the meaning of alpha smaller than -1 in fig2?

Reply – The values of w_{ij}^{L} and w_{ij}^{G} are non-negative, and those of $(\alpha_{\max} - \alpha)$ and $(1 + \alpha)$ are non-negative in the range $-1 < \alpha < \alpha_{\max}$. Therefore, the weights $-(\alpha_{\max} - \alpha)w_{ij}^{\mathrm{L}}$ and $-(1 + \alpha)w_{ij}^{\mathrm{G}}$ are non-positive and represent inhibitory effects

between neurons. This interpretation, however, is valid only in the above value range, so models with $\alpha < -1$ are not biologically meaningful as the reviewer doubted. Nevertheless, we performed simulations for α<-1 to check the mathematical properties of the model in the whole range of α. With those simulations, we could validate the results of our mathematical analysis, which suggests that meaningful memory recall occurs only in the range α>-1 in agreement with the biologically meaningful range of the parameter.

7. If I understand it correctly, this novelty index is a way to set the adjacency matrix of the graph and as such set H? Could you elaborate on s(mu,nu) used in the methods in equation 30?

Reply – We are afraid of whether we accurately understood the point raised by the reviewer. The index $s(\mu, \nu)$ merely represents the correlation between two attractor patterns triggered by patterns μ and ν when we used LAM, or represents the cosine similarity between two low-dimensional representation vectors when we used the Laplacian eigenmaps (namely, the embedding of nodes using GL eigenvectors with low eigenvalues). To avoid confusion, we have revised the explanation of the novelty index in the Methods section (L805 – L819). We hope the revised description aids the understanding of the method.

--

Reviewer #2: Summary:

In ``Multiscale representations of community structures in attractor neural networks,'' the authors study the brain's ability to represent hierarchical structure through the use of Laplacian associative memory (LAM). The authors study three commonly used graphs: a 4-regular graph used in graph learning experiments, a Karate-club network, and a compartmentalized room network. First, the authors introduce LAMs, which are a generalized form of Hopfield networks with hetero-associative weights. The authors then use the the three commonly used graph types as the hetero-associative weights, and demonstrate that the strength of auto-association (determined by $¥alpha$) tunes the representational overlap of LAM attractors. Next, the authors rewrite the Hopfield energy function as a function of two competing terms: $¥tilde{m}^T L ¥tilde{m}$ and $¥tilde{m}^T D ¥tilde{m}$ and demonstrate that LAM dynamics balance an optimization between

the conventional Hopfield cost, $¥tilde{m}^T D ¥tilde{m}$, and the pattern overlap cost, $¥tilde{m}^T L ¥tilde{m}$. The authors find that the overlapped pattern (as determined by $¥alpha$) is well explained by the eigenvectors of the smallest eigenvalues of the graph Laplacian. Finally, the authors generalize the utility of their findings by using LAMs to detect graph bottlenecks, perform image segmentation, and chunk sequential activity in time for asymmetric LAMs.

Overall, I find this work to be quite interesting and impactful. While the role of graph Laplacians in detecting community structure is well studied, there is increasing interest in the neural mechanisms that underlie our mental representations of community structure. This work provides an elegant and intuitive candidate mechanism using an extension of the very well-known Hopfield model. However, there are several major concerns I have about methodology and presentation that would need to be addressed before recommending acceptance.

Reply – We thank the reviewer for the valuable comments on our manuscript.

Comments:

- Generalizability of results to parameters: My first main concern is how generalizable these findings are to different choices of simulation parameters. I appreciate the authors' analytical relationship between the LAM energy function and graph Laplacian. However, the numerical validation of the theory, as far as I can tell, consist of a few simulations in a very specific parameter regime. I would like to see how robustly the results of Figures 2 and 3 behave under different numbers of neurons (N) and representational sparsities (p).

Reply – We have performed additional simulations with different values of N (the number of neurons) and p (sparsity). We have summarized these results at L219 – L224 in the main text and also in Figs. S2 and S3. Basically, N should be large enough to get good performance (like the performance of memory recall in the conventional Hopfield model. However, the model behavior (i.e., the correlation structure of attractor patterns) for the four-room graph was qualitatively unchanged in the range N >=5000. Regarding the parameters p, changing their values around the original values did not significantly change the model behavior. These results indicate that the performance of LAM is robust against changes in parameter values within a reasonable range.

- Generalizability of results to other hierarchical random graphs: My second main concern is how generalizable these findings are to more complex hierarchical structure. While I very much appreciate the authors' selection of relevant graph types, I would like to see reproductions of the results of Figures 2 and 3 on other common models of hierarchical graphs. My motivation for this question is that in Figures 2 and 3, the authors only use 3 specific instances of graphs, where two of them (4-regular graph and compartmentalized room) have very regular connectivities and simple hierarchies. In contrast, real neural systems have to identify hierarchical structure in a distribution of graphs that are often irregular. Hence, I would like to see Figures 2 and 3 replicated on a family of hierarchical random graphs where you can change the number of nodes and communities, and measure 1) the effect of graph size (as measured by number of nodes), 2) the effect of randomness in graph connectivity (due to randomness in different instantiations of the hierarchical random graphs), and 3) the effect of number of communities and levels of hierarchy. Of particular importance is #3, because the three graphs that the authors currently study do not have many levels of hierarchy. The 4-regular graph and compartmentalized room structure both only have one level of hierarchy (interconnected modules), and the karate-club network is quite small.

Reply – We performed additional simulations in the revised manuscript, in which we systematically generated the hierarchical community structures of random graphs by using a stochastic block model. We simulated our model on random graphs with various numbers of nodes, communities, and hierarchies, and different choices of random seeds (to generate the different realization of graph structure). We summarized these results at L315 – L345 in the main text and also in Figs. 4, S7, and S8. We observed the same tendency across the random graphs simulated in this study. We hope these novel results alleviate the reviewer's concern about the generalizability of our model.

- Presentation of methods: I found it quite difficult to read through the results, because many modeling choices and expressions were not described. Upon reading the methods, many of my questions were answered. I highly recommend that the authors reference the specific section of the methods by name that are relevant in the results. Some examples are:
= The derivation of Eq. 2, and subsequent definitions of terms such as $¥tilde{¥xi}$ and $V = p(1-p)$,

= The activity-dependent learning rule and its convergent form
= The decomposition of w_{ij} into excitatory and inhibitory synaptic weights
= The pattern overlap vector in rewriting the energy function
This list is not exhaustive, but are the main parts of the paper that I found most difficult to understand. For example, I recommend the authors state something to the effect of ``(for more details about the derivation of Eq. 3, see methods: Decomposition of excitatory and inhibitory synaptic weights)'' after equation 3.

Reply – Following the suggestions by the reviewer, we have made the following modifications in the revised manuscript. Please note we have eliminated the on-line learning rule and its derivations according to the comment 1 of the reviewer #1:
= We have moved the definitions of hetero-associative weights, decomposed weights, and pattern overlap vectors from the Methods to the Results (L116 - L124, L147 - L150, and L264).
= We have unambiguously related the corresponding descriptions between the Methods and the Results (L209, L241, L275, L286, L319, L372, and L501).

- Methodological details: I would like to see the number of neurons and sparsity ratio moved from the methods to the results. As I was reading, I would have found it intuitive and helpful for this information to be present early on in the results.

Reply – Following the suggestion, we have moved the description of parameter values from the Methods to the Results (L104).

- Proof of monotonic energy decrease: The authors demonstrate in Figure S2 that the energy decreases over time. While the claim that equations 5 and 6 serve as energy functions for the LAMs are fine in the context of Figure S2, is this generally true? It is a very strong statement to say that Eq. 5 is truly and globally an energy function for the LAM. Is this statement mathematically provable?

Reply – In the Hopfield-type associative memory models, a monotonic decrease of the energy function is rigorously guaranteed if the synaptic matrix is symmetric, and the state of each neuron undergoes an asynchronous sequential update (Hopfield, 1982). To accelerate the simulations, we used a synchronous parallel update in this study. Practically, however, it is known that the parallel update of neural activities does not significantly change the behavior of the model. Actually, in the mean-field approximation,

both sequential update and parallel update settle into the same dynamical equation for population activity (c.f. Haga & Fukai, PRL, 2019). However, since a monotonic decrease is not rigorous in our model, we have revised the manuscript to clarify this point (L259-263).

- Rigor of notation and derivations: Several times in the text, I noticed the usage of equal signs when approximation signs should have been used. This is seen in Figure 3D where the pattern overlap is an approximate sum of the 2nd, 3rd, and 4th GL eigenvectors, but an equality is used. Another example is the statement in 192: ``As in the conventional Hopfield model, dynamics of LAM monotonically decrease this energy.'' It is unclear whether this statement is generally true, or only true for these particular examples. In general, I believe the results and methods would benefit from more care in explicitly stating the conditions for which they are true.

Reply – We apologize the lack of rigor in our expression. We have corrected the schematics in Figs. 1 and 3D and descriptions about the monotonic decrease of the energy function as mentioned above.

- Clarity of methodological derivation: While I appreciate the derivations in the results and methods, they are quite difficult to follow. I would like the authors to expand upon the derivations in the methods so that they can be more easily followed. Some specific sections that I would like to see extra explanations for are:
= Construction of hetero-associative weights. In particular, there are a lot of operations that are performed between equations 8-11. Could the authors break up the derivation into more intermediary parts, and describe using words exactly what they did (for example, exactly which variables were substituted)?
= Decomposition of excitatory and inhibitory synaptic weights. In particular, equations 12-15 are simply presented, without any explanation for why they are defined that way.
= Analysis of the energy function of LAM. Could the authors write a few sentences about what a ``pattern overlap vector'' is? I understand how it is defined in equation 17, but it would help to understand, for example, why the authors call it a ``pattern overlap vector.''

Reply – We apologize the lack of clarity in the previous manuscript. We have made the following changes:

= Eqs. (8)-(11) were deleted to avoid confusion (according to the comment 1 of the reviewer #1).
= Decomposition of excitatory and inhibitory weights were moved from the Methods to the Results, where the interpretations of these weights are also described (L143-L161).
= Pattern overlap is an index of memory recall that measures the degree of overlap between an embedded memory pattern and recalled activity patterns. This index has been traditionally used in the analysis of the Hopfield-type model because its macroscopic dynamics and energy function can be written in terms of pattern overlaps. We have described this point in the revised manuscript (L180-L184). Furthermore, we elaborated on the derivations of the energy function of LAM in the Methods section (L672 – L685).

- Parameter choices: Why do the authors change the number of neurons (N=30,000 for image segmentation, N=10,000 for the rest), the time step ($¥eta = 0.01$ for symmetric simulations, and $¥eta = 0.1$ for sequences), and $¥gamma = 0.6$ for the image segmentation, and $¥gamma = 0.3$ for the rest? Is it possible to find a single parameter regime where all behaviors are observed? While it is not crucial that the authors can find such a regime, it would improve confidence in the generalizability of the results.

Reply – As we show in the additional simulations, N must be large enough to get stable performance. The demanded number depends on the number of embedded patterns as in the conventional Hopfield models. For image segmentation tasks, we embedded the larger number of patterns (corresponding to the number of pixels) than other simulations, so we had to increase the number of neurons. We could use N=30000 setting for all simulations but it would unreasonably increase computational time especially when we simulate the model with various parameter settings.

The pitch of discretization η could also be set to 0.01 in all simulations, but we used 0.1 for asymmetric LAM to accelerate the simulations. This large value could have increased noise to impair the stability of attractors. However, the particular value did not cause any problem in the present simulations of sequential retrieval dynamics.

In contrast with N and η, it was hard to determine a single suitable value of γ common to all cases. In our model, the additional global inhibition term helps to generate sparse representations for large values of α, but too strong inhibition obviously impairs the

model behavior. The optimal value of γ depends on the task and the choices of other parameters, but we have not completely understood this dependence. Therefore, we manually tuned these values in this work. The analysis of optimal parameter settings in LAM is an interesting open question that we wish to address in future studies. We discussed this point in the Discussion section (L561 – L566).

Reviewer #3: LAM Review

in this paper, the authors propose the Laplacian associative memory (LAM) model, an extension of Hopfield networks which additionally allows associations between different memory patterns (hetero-associations). They show empirically that this allows LAM to discover clusters of associated memories (community structure) at different scales, which has been previously demonstrated in humans and animals in behavioral and neural studies. Relatedly, they also show that LAM can also detect subgoals as bottlenecks between clusters. They also demonstrate chunked sequential activation patterns similar to hippocampal theta sequences. Finally, the authors draw explicit links between the graph Laplacian and LAM. Specifically, they show theoretically that, under certain assumptions, the energy function minimized LAM is equivalent to the objective function of graph segmentation, what can be optimized by taking the eigenvectors of the graph Laplacian. It is also noteworthy that they derive a biologically plausible implementation of LAM, which allows them to trace some of the effects to the parameter alpha which controls the balance between global and local inhibition. It's especially cool how this balance controls the scale of the discovered clusters.

How the brain organizes the world hierarchically is a central question in neuroscience. This paper brings us closer to an answer by showing how graph clustering could be implemented in a biologically plausible circuit and confirming that it exhibits activation patterns similar to those found in neural data. The paper is written clearly and is relatively straightforward to follow, and provides a pleasant mix of theoretical analyses and simulations of neural and behavioral data. I think the paper could be published in its current form, so I'm going to give some relatively minor suggestions.

Reply - We thank the reviewer for the encouraging comments.

1. It's a little unclear to me whether LAM is really conceptually different from associative models for learning temporal sequences. Specifically, one way to learn the structure of the graph is by doing a random walk (or some other kind of traversal) and establishing connections between successive stimuli. Indeed, this is how humans in the Schapiro et al. task learn the graph, and also how the RNN they propose in one of their papers learns it too. The one-dimensional chain case is a special case of this, but I don't see why, if a temporal sequence model traverses a graph (so visiting the same state for a second time would elicit the same memory pattern), it wouldn't also pick up on its community structure (in fact, if I recall correctly, this is exactly what Schapiro's RNN does). If the distinction is that for LAM is that the order of presentation of the memory patterns is irrelevant, then that should be highlighted, as opposed to the one-dimensional chain special case.

Reply – As the reviewer pointed out, our model is potentially applicable to non-sequential presentations of memory items. The model is applicable if the structure of hetero-association and inhibitory connections are created by any learning scheme that can create the symmetric Hebbian connection matrix hypothesized in this study. Though we did not specify a particular learning mechanism, we assumed that neural circuits in the brain learn the statistical structure of episodes through sequential experiences, as supported by the existence of such strategy in the human brain (probably also in the brain of monkeys and rats). As doubted by the reviewer, our model may not be novel in that aspect. However, the main finding of this study resides in the recall process of hierarchical memory structures rather than the learning process. Especially, our model offers the flexible modulation of the scale of representations during the recall of community structures. Importantly, the representation scale needs not to be specified during the learning process. To our knowledge, no previous models provide this flexible recall of hierarchical community structures at multiple representational scales. Therefore, we believe that our model contains essential differences from the previous models even though these models were built in the same spirit.

We agree with the interpretation of "one-dimensional chain" that all sequential experiences are essentially one-dimensional chain of inputs. However, this does not necessarily imply that the structure behind sequential experiences is also one-dimensional. For example, in Shapiro's experiment, sequences of sensory inputs were generated by random walk on a complex graph. Each sequential experience was a one-

dimensional chain of items, but the underlying graph structure was not one-dimensional. In contrast, the previous associative memory network model by Griniasty et al. (1993) was structurally one-dimensional because it modeled an experiment in which a fixed sequence of sensory inputs was repeated (Miyashita, 1989). This experiment is equivalent to sensory experience during going back and forth on the same one-dimensional track, but Schapiro's experiment is essentially different. We extended the associative memory network model for experiences on a one-dimensional chain to experiences on more general graph structures like Schapiro's experiment. To clarify this point, we have revised the related descriptions in the Results section (L136-139).

2. The interpretation of the sign of alpha throughout the paper seems a bit confusing without considering alpha_max or gamma – e.g. from Eq. 3, if alpha_max is very large, alpha = -1 means only local inhibition, but alpha = 0 means mostly local inhibition. Also in equation 2, the effect of alpha clearly depends on gamma. Basically, it's unclear to me if the setpoint of zero carries any special significance (this is also evident in figure 2).

Reply – We agree with the point raised by the reviewer. We regarded zero as a threshold because one of the major novel points of our work is considering a negative region of alpha. However, both theoretically and empirically, $\alpha=0$ is not a phase transition point for the model. Following the reviewer's suggestion, we have revised the related descriptions in the Results section (L206) and modified Figure 1.

3. Relatedly, and the results section, it would be useful to clarify how the interplay between global and local inhibition produces the results, e.g. in Fig. 2. An intuitive explanation would suffice.

Reply – Generally, $\alpha\approx-1$ corresponds to a local-inhibition-dominant state in which the model retrieves a mixed memory representation while a large positive value of α corresponds to a global-inhibition-dominant state in which the model sparsely recalls memory patterns as in the conventional Hopfield model. We have stated these points in the Results section (L203 and L207).

4. Some results in the paper, in particular figures 4, 5, and 6 are largely qualitative. It will be useful if the authors compute relevant summary statistics and perform the corresponding statistical tests.

Reply – In the previous studies of subgoal findings, the definition of subgoals by itself depended on the graph Laplacian (e.g., Simsek et al., 2005; Machado et al., 2017). There is no ground truth in such a case, and therefore these studies did not attempt to evaluate the accuracy of subgoal findings. To evaluate the goodness of subgoals, we should investigate, for example, the performance of reinforcement learning based on the subgoals identified by LAM. This is an ongoing project of our laboratory, and we will report the results in the future.

We showed the simulation results about theta sequences for comparison with experimental data (Fig. 7 in Gupta et al., 2012), but this experimental study did not provide summary statistics and statistical tests. Our current simulation settings were also simplified compared to the experimental settings. Therefore, we only showed qualitative matches in the present study.

5. Relatedly, it would be useful if if the authors put side-by-side neural data next to the simulation results in Fig 6.

Reply – Following the suggestion by the reviewer, we have put the experimental data (Fig. 7 in Gupta et al., 2012) side-by-side with our simulation results (Fig. 8L in the revised manuscript). We feel that this modification has made the biological relevance of the model clearer. We thank the reviewer for the advice.

6. in the bottleneck and the overrepresentation model, the additional nodes do not have the their own simulated patterns but are rather set artificially (line 591). I understand why this would be convenient for computational tractability, but it seems a bit misleading since the emergent similarity of the attractor states corresponding to those patterns is crucial for the result.

Reply – We are afraid of whether we could correctly understand the point of this comment. We would like to mention that the additional nodes had their own simulated patterns. We assigned independent memory patterns to the additional nodes at the bottleneck and over-representation and included activity patterns on these nodes in our estimations of pattern overlaps. A major difference between the nodes in the central ring and the additional nodes of the maze was whether they received external stimuli or not. However, every additional node was at least connected to one of the core nodes, and

both of them belonged to the same community. Therefore, the nodes in the central ring and the additional nodes exhibited similar activity patterns, implying that whether the additional nodes are stimulated does not significantly change the simulation results.

A few grammatical errors in the introduction:

L. 67 – correlations depend
L. 60 – their, mechanism
L. 83 – call our model as
L. 88 — with asymmetric
L. 90 – for hierarchical

Reply – We thank the reviewer for pointing out these errors. We have corrected them in the revised manuscript. We asked a professional proofreading service to check our manuscript before the resubmission.
